# Methyl 2-ethyl-4-hydr­oxy-2*H*-1,2-benzo­thia­zine-3-carboxyl­ate 1,1-dioxide

**DOI:** 10.1107/S1600536810012365

**Published:** 2010-04-14

**Authors:** Muhammad Nadeem Arshad, Muhammad Zia-ur-Rehman, Islam Ullah Khan

**Affiliations:** a Materials Chemistry Laboratory, Department of Chemistry, GC University, Lahore, Pakistan; b Applied Chemistry Research Centre, PCSIR Laboratories Complex, Lahore 54600, Pakistan

## Abstract

In the title compound, C_12_H_13_NO_5_S, the thia­zine ring adopts a half chair conformation and an intra­molecular O—H⋯O hydrogen bond generates an S(6) ring. In the crystal, the mol­ecules are linked by C—H⋯O inter­actions, leading to zigzag chains along the *b* axis.

## Related literature

For background to the biological properties of thia­zines, see: Zia-ur-Rehman *et al.* (2005 ▶, 2006 ▶). For related structures, see: Arshad *et al.* (2009*a* ▶,*b* ▶). For graph-set notation, see: Bernstein, *et al.* (1995 ▶).
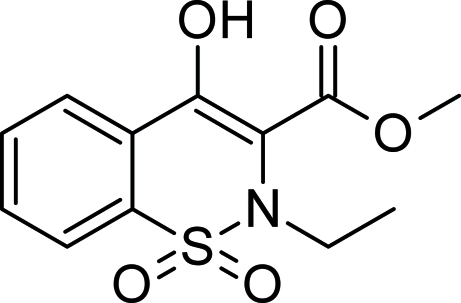


## Experimental

### 

#### Crystal data

C_12_H_13_NO_5_S
*M* *_r_* = 283.29
Orthorhombic, 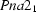

*a* = 7.2460 (6) Å
*b* = 20.548 (2) Å
*c* = 8.5710 (8) Å
*V* = 1276.1 (2) Å^3^
*Z* = 4
Mo *K*α radiation
μ = 0.27 mm^−1^
*T* = 296 K
0.24 × 0.14 × 0.10 mm

#### Data collection

Bruker KAPPA APEXII CCD diffractometer
Absorption correction: multi-scan (*SADABS*; Bruker, 2007 ▶) *T*_min_ = 0.938, *T*_max_ = 0.974
14253 measured reflections
3148 independent reflections
1270 reflections with *I* > 2σ(*I*)
*R*_int_ = 0.133

#### Refinement

*R*[*F* ^2^ > 2σ(*F* ^2^)] = 0.054
*wR*(*F* ^2^) = 0.102
*S* = 0.95
3148 reflections
176 parameters
1 restraint
H-atom parameters constrained
Δρ_max_ = 0.22 e Å^−3^
Δρ_min_ = −0.25 e Å^−3^
Absolute structure: Flack (1983 ▶), 1464 Friedel pairs
Flack parameter: 0.07 (12)

### 

Data collection: *APEX2* (Bruker, 2007 ▶); cell refinement: *SAINT* (Bruker, 2007 ▶); data reduction: *SAINT*; program(s) used to solve structure: *SHELXS97* (Sheldrick, 2008 ▶); program(s) used to refine structure: *SHELXL97* (Sheldrick, 2008 ▶); molecular graphics: *ORTEP-3* (Farrugia, 1997 ▶) and *PLATON* (Spek, 2009 ▶); software used to prepare material for publication: *X-SEED* (Barbur, 2001 ▶); *WinGX* (Farrugia, 1999 ▶) and *PLATON*.

## Supplementary Material

Crystal structure: contains datablocks I, New_Global_Publ_Block. DOI: 10.1107/S1600536810012365/hb5390sup1.cif

Structure factors: contains datablocks I. DOI: 10.1107/S1600536810012365/hb5390Isup2.hkl

Additional supplementary materials:  crystallographic information; 3D view; checkCIF report

## Figures and Tables

**Table 1:** Hydrogen-bond geometry (Å, °)

*D*—H⋯*A*	*D*—H	H⋯*A*	*D*⋯*A*	*D*—H⋯*A*
O3—H3⋯O4	0.82	1.92	2.628 (5)	144
C4—H4⋯O4^i^	0.93	2.55	3.395 (5)	152
C10—H10*B*⋯O2^ii^	0.96	2.52	3.320 (5)	141

Symmetry codes: (i) 
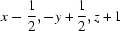
; (ii) 
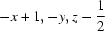
.

**Fig. 1.**
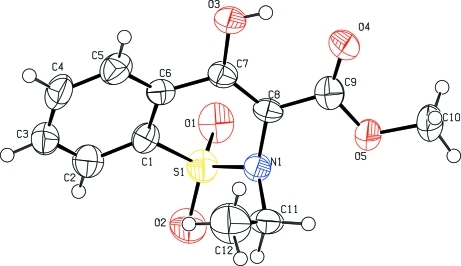
The molecular structure of (I) showing 50% displacement ellipsoids.

**Fig. 2.**
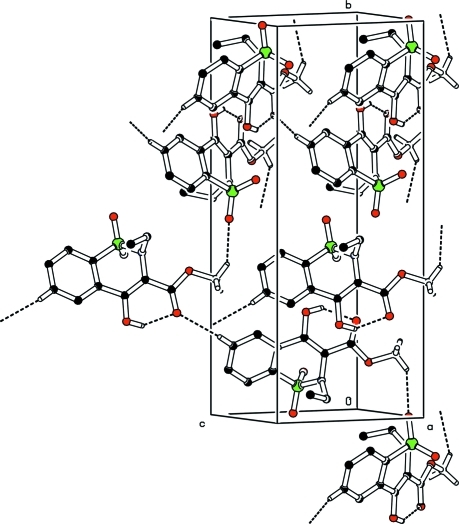
Unit cell packing for (I) showing the inter and intramolecular hydrogen bondings using dashed lines. Hydrogen atoms have been omitted for clarity.

## supplementary crystallographic information

### Comment

Under the heading of synthesis and X-ray studies and biological evaluation of thiazine related heterocycles our group has already reported biological applications (Zia-ur-Rehman *et al.*, 2005, 2006) and the crystal structures of 1,2-benzothiazine derivatives (Arshad *et al.*, 2009*a*,*b*) II & III. The title compound Methyl 2-ethyl-4-hydroxy-2*H*-1,2-benzothiazine-3-carboxylate 1,1-dioxide (I) is different only in *H*-alkylation. The hydrogen bonding interactions in I, II and III are pretty much common. The intramolecular O–H···O interaction tend to rise the six membered ring motif R_1_^1^(6) (Bernstein, *et al.*, 1995) which is almost planar with the r.m.s deviaton of 0.0156Å and iclined at dihedral angle of 25.1 (6)° & 17.5 (6)° with respect to the thiazine and benzene ring respectively. The half chair shaped thiazine ring exihibits a maximum deviation from the least square plane measure 0.347 (2)Å for S1 and 0.345 (2)Å for C1.  The intermoleculear C–H···O hydrogen bonding forms zig-zag network along the *b* axes. The bond lngths and bond angles are compareable with the molecules II and III.

### Experimental

Ethyl iodide (250 mg, 1.6 mmol) was added drop wise to the mixture of methyl 4-hydroxy-2H-1,2-benzothiazine-3-carboxylate-1,1-dioxide (350 mg, 1.37 mmol), anhydrous potassium carbonate (161 mg, 1.6 mmol) and dimethylformamide (5 ml) in a round bottom flask. Contents were stirred at room temperature for 5 h under nitrogen atmosphere and poured over ice cooled water (100 ml) resulting white precipetates, which was filtered and washed with cold water.  Colourless needles of (I) were obtained by re-crystallization from a methanol solution under slow evaporation.

### Crystal data

**Table:**

C_12_H_13_NO_5_S	*F*(000) = 592
*M**_r_* = 283.29	*D*_x_ = 1.475 Mg m^−^^3^
Orthorhombic, *P**n**a*2_1_	Mo *K*α radiation, λ = 0.71073 Å
Hall symbol: P 2c -2n	Cell parameters from 733 reflections
*a* = 7.2460 (6) Å	θ = 2.6–17.3°
*b* = 20.548 (2) Å	µ = 0.27 mm^−^^1^
*c* = 8.5710 (8) Å	*T* = 296 K
*V* = 1276.1 (2) Å^3^	Cut needle, colourless
*Z* = 4	0.24 × 0.14 × 0.10 mm

### Data collection

**Table:**

Bruker KAPPA APEXII CCD diffractometer	3148 independent reflections
Radiation source: fine-focus sealed tube	1270 reflections with *I* > 2σ(*I*)
graphite	*R*_int_ = 0.133
φ and ω scans	θ_max_ = 28.3°, θ_min_ = 2.0°
Absorption correction: multi-scan (*SADABS*; Bruker, 2007)	*h* = −9→9
*T*_min_ = 0.938, *T*_max_ = 0.974	*k* = −26→27
14253 measured reflections	*l* = −11→11

### Refinement

**Table:**

Refinement on *F*^2^	Hydrogen site location: inferred from neighbouring sites
Least-squares matrix: full	H-atom parameters constrained
*R*[*F*^2^ > 2σ(*F*^2^)] = 0.054	*w* = 1/[σ^2^(*F*_o_^2^) + (0.0219*P*)^2^] where *P* = (*F*_o_^2^ + 2*F*_c_^2^)/3
*wR*(*F*^2^) = 0.102	(Δ/σ)_max_ < 0.001
*S* = 0.95	Δρ_max_ = 0.22 e Å^−^^3^
3148 reflections	Δρ_min_ = −0.25 e Å^−^^3^
176 parameters	Extinction correction: *SHELXL*, Fc^*^=kFc[1+0.001xFc^2^λ^3^/sin(2θ)]^-1/4^
1 restraint	Extinction coefficient: 0.0062 (9)
Primary atom site location: structure-invariant direct methods	Absolute structure: Flack (1983), 1464 Friedel pairs
Secondary atom site location: difference Fourier map	Flack parameter: 0.07 (12)

### Special details

**Table:**

Geometry. All s.u.'s (except the s.u. in the dihedral angle between two l.s. planes) are estimated using the full covariance matrix. The cell s.u.'s are taken into account individually in the estimation of s.u.'s in distances, angles and torsion angles; correlations between s.u.'s in cell parameters are only used when they are defined by crystal symmetry. An approximate (isotropic) treatment of cell s.u.'s is used for estimating s.u.'s involving l.s. planes.
Refinement. Refinement of F^2^ against ALL reflections. The weighted R-factor wR and goodness of fit S are based on F^2^, conventional R-factors R are based on F, with F set to zero for negative F^2^. The threshold expression of F^2^ > 2σ(F^2^) is used only for calculating R-factors(gt) etc. and is not relevant to the choice of reflections for refinement. R-factors based on F^2^ are statistically about twice as large as those based on F, and R- factors based on ALL data will be even larger.

### Fractional atomic coordinates and isotropic or equivalent isotropic displacement parameters (Å^2^)

**Table:**

	*x*	*y*	*z*	*U*_iso_*/*U*_eq_	
S1	0.19292 (16)	0.06952 (6)	0.49242 (15)	0.0514 (4)	
O1	0.0578 (4)	0.10023 (16)	0.3936 (4)	0.0648 (10)	
O2	0.1809 (4)	0.00107 (13)	0.5198 (4)	0.0716 (10)	
O3	0.3931 (4)	0.26033 (12)	0.5272 (4)	0.0532 (8)	
H3	0.4444	0.2709	0.4458	0.080*	
O4	0.5638 (4)	0.23848 (15)	0.2618 (4)	0.0563 (10)	
O5	0.5818 (4)	0.13380 (14)	0.1843 (4)	0.0550 (9)	
N1	0.3974 (4)	0.08716 (17)	0.4241 (4)	0.0413 (9)	
C1	0.1976 (6)	0.1103 (2)	0.6695 (5)	0.0422 (11)	
C2	0.1285 (6)	0.0824 (2)	0.8054 (6)	0.0536 (14)	
H2	0.0732	0.0416	0.8027	0.064*	
C3	0.1429 (6)	0.1160 (3)	0.9446 (6)	0.0560 (15)	
H3A	0.1030	0.0970	1.0370	0.067*	
C4	0.2163 (6)	0.1776 (2)	0.9459 (5)	0.0589 (15)	
H4	0.2201	0.2009	1.0389	0.071*	
C5	0.2849 (6)	0.2056 (2)	0.8105 (6)	0.0560 (14)	
H5	0.3341	0.2474	0.8139	0.067*	
C6	0.2811 (6)	0.1722 (2)	0.6708 (6)	0.0396 (11)	
C7	0.3701 (5)	0.1955 (2)	0.5296 (6)	0.0406 (11)	
C8	0.4294 (6)	0.1557 (2)	0.4160 (5)	0.0372 (11)	
C9	0.5296 (6)	0.1805 (3)	0.2822 (5)	0.0471 (12)	
C10	0.6951 (6)	0.1538 (2)	0.0543 (5)	0.0707 (16)	
H10A	0.6288	0.1848	−0.0079	0.106*	
H10B	0.7252	0.1165	−0.0083	0.106*	
H10C	0.8066	0.1733	0.0926	0.106*	
C11	0.5572 (6)	0.0441 (2)	0.4634 (5)	0.0548 (15)	
H11A	0.6478	0.0475	0.3805	0.066*	
H11B	0.5143	−0.0006	0.4657	0.066*	
C12	0.6504 (6)	0.0588 (3)	0.6154 (7)	0.0840 (19)	
H12A	0.6928	0.1031	0.6152	0.126*	
H12B	0.7537	0.0301	0.6290	0.126*	
H12C	0.5646	0.0527	0.6994	0.126*	

### Atomic displacement parameters (Å^2^)

**Table:**

	*U*^11^	*U*^22^	*U*^33^	*U*^12^	*U*^13^	*U*^23^
S1	0.0554 (7)	0.0474 (7)	0.0516 (7)	−0.0144 (7)	0.0019 (9)	−0.0055 (8)
O1	0.051 (2)	0.083 (3)	0.060 (2)	−0.0095 (18)	−0.010 (2)	−0.003 (2)
O2	0.097 (2)	0.0399 (19)	0.078 (3)	−0.0283 (16)	0.014 (2)	−0.010 (2)
O3	0.065 (2)	0.033 (2)	0.061 (3)	−0.0045 (14)	0.0097 (19)	0.0007 (19)
O4	0.067 (2)	0.042 (2)	0.059 (2)	−0.0064 (17)	0.0121 (19)	0.0070 (18)
O5	0.068 (2)	0.048 (2)	0.050 (2)	0.0000 (17)	0.0208 (19)	−0.0001 (19)
N1	0.044 (2)	0.037 (2)	0.043 (2)	−0.0036 (17)	0.0052 (18)	−0.0020 (18)
C1	0.038 (3)	0.048 (3)	0.040 (3)	0.000 (2)	0.000 (2)	−0.006 (3)
C2	0.038 (3)	0.056 (4)	0.066 (4)	−0.003 (2)	0.009 (3)	0.005 (3)
C3	0.056 (3)	0.062 (4)	0.050 (4)	0.010 (3)	0.012 (3)	0.008 (3)
C4	0.062 (3)	0.075 (4)	0.039 (4)	0.017 (3)	0.006 (3)	−0.010 (3)
C5	0.061 (3)	0.051 (3)	0.056 (4)	0.002 (3)	0.003 (3)	−0.011 (3)
C6	0.039 (3)	0.035 (3)	0.045 (3)	0.002 (2)	−0.001 (3)	0.000 (3)
C7	0.044 (3)	0.033 (3)	0.045 (3)	−0.005 (2)	−0.003 (2)	−0.001 (3)
C8	0.040 (3)	0.027 (3)	0.045 (3)	−0.004 (2)	−0.005 (2)	0.002 (2)
C9	0.043 (3)	0.058 (3)	0.040 (3)	−0.003 (3)	−0.006 (3)	−0.001 (3)
C10	0.086 (4)	0.065 (4)	0.061 (4)	−0.004 (3)	0.036 (3)	0.002 (3)
C11	0.057 (3)	0.037 (3)	0.070 (4)	0.010 (2)	0.005 (3)	0.010 (3)
C12	0.069 (4)	0.102 (5)	0.081 (5)	0.019 (3)	−0.013 (3)	0.015 (4)

### Geometric parameters (Å, °)

**Table:**

S1—O2	1.429 (3)	C4—C5	1.388 (6)
S1—O1	1.441 (3)	C4—H4	0.9300
S1—N1	1.634 (3)	C5—C6	1.381 (6)
S1—C1	1.734 (5)	C5—H5	0.9300
O3—C7	1.343 (4)	C6—C7	1.452 (6)
O3—H3	0.8200	C7—C8	1.343 (5)
O4—C9	1.229 (5)	C8—C9	1.450 (6)
O5—C9	1.330 (5)	C10—H10A	0.9600
O5—C10	1.444 (5)	C10—H10B	0.9600
N1—C8	1.428 (5)	C10—H10C	0.9600
N1—C11	1.495 (5)	C11—C12	1.499 (6)
C1—C2	1.392 (6)	C11—H11A	0.9700
C1—C6	1.408 (5)	C11—H11B	0.9700
C2—C3	1.383 (6)	C12—H12A	0.9600
C2—H2	0.9300	C12—H12B	0.9600
C3—C4	1.372 (6)	C12—H12C	0.9600
C3—H3A	0.9300		
			
O2—S1—O1	119.1 (2)	C1—C6—C7	118.8 (4)
O2—S1—N1	109.44 (18)	C8—C7—O3	123.6 (4)
O1—S1—N1	107.97 (19)	C8—C7—C6	123.0 (4)
O2—S1—C1	109.4 (2)	O3—C7—C6	113.3 (4)
O1—S1—C1	108.4 (2)	C7—C8—N1	120.9 (4)
N1—S1—C1	100.9 (2)	C7—C8—C9	121.3 (4)
C7—O3—H3	109.5	N1—C8—C9	117.9 (4)
C9—O5—C10	116.3 (3)	O4—C9—O5	123.6 (4)
C8—N1—C11	117.9 (3)	O4—C9—C8	123.7 (4)
C8—N1—S1	112.5 (3)	O5—C9—C8	112.7 (4)
C11—N1—S1	119.4 (3)	O5—C10—H10A	109.5
C2—C1—C6	121.3 (4)	O5—C10—H10B	109.5
C2—C1—S1	121.8 (4)	H10A—C10—H10B	109.5
C6—C1—S1	116.9 (4)	O5—C10—H10C	109.5
C3—C2—C1	119.3 (4)	H10A—C10—H10C	109.5
C3—C2—H2	120.4	H10B—C10—H10C	109.5
C1—C2—H2	120.4	N1—C11—C12	115.2 (4)
C4—C3—C2	119.8 (5)	N1—C11—H11A	108.5
C4—C3—H3A	120.1	C12—C11—H11A	108.5
C2—C3—H3A	120.1	N1—C11—H11B	108.5
C3—C4—C5	120.9 (4)	C12—C11—H11B	108.5
C3—C4—H4	119.5	H11A—C11—H11B	107.5
C5—C4—H4	119.5	C11—C12—H12A	109.5
C6—C5—C4	120.8 (4)	C11—C12—H12B	109.5
C6—C5—H5	119.6	H12A—C12—H12B	109.5
C4—C5—H5	119.6	C11—C12—H12C	109.5
C5—C6—C1	117.7 (4)	H12A—C12—H12C	109.5
C5—C6—C7	123.3 (4)	H12B—C12—H12C	109.5
			
O2—S1—N1—C8	−171.2 (3)	S1—C1—C6—C7	−4.9 (5)
O1—S1—N1—C8	57.8 (3)	C5—C6—C7—C8	153.4 (4)
C1—S1—N1—C8	−55.9 (3)	C1—C6—C7—C8	−21.9 (6)
O2—S1—N1—C11	−26.6 (4)	C5—C6—C7—O3	−24.2 (6)
O1—S1—N1—C11	−157.6 (3)	C1—C6—C7—O3	160.5 (4)
C1—S1—N1—C11	88.7 (3)	O3—C7—C8—N1	−178.8 (4)
O2—S1—C1—C2	−22.5 (4)	C6—C7—C8—N1	3.8 (6)
O1—S1—C1—C2	108.9 (4)	O3—C7—C8—C9	2.0 (6)
N1—S1—C1—C2	−137.8 (4)	C6—C7—C8—C9	−175.4 (4)
O2—S1—C1—C6	155.2 (3)	C11—N1—C8—C7	−105.7 (4)
O1—S1—C1—C6	−73.4 (4)	S1—N1—C8—C7	39.5 (5)
N1—S1—C1—C6	39.9 (4)	C11—N1—C8—C9	73.6 (5)
C6—C1—C2—C3	−0.3 (7)	S1—N1—C8—C9	−141.2 (3)
S1—C1—C2—C3	177.3 (3)	C10—O5—C9—O4	4.3 (7)
C1—C2—C3—C4	3.3 (7)	C10—O5—C9—C8	−175.3 (4)
C2—C3—C4—C5	−3.2 (7)	C7—C8—C9—O4	−1.5 (7)
C3—C4—C5—C6	0.0 (7)	N1—C8—C9—O4	179.3 (4)
C4—C5—C6—C1	2.9 (6)	C7—C8—C9—O5	178.1 (4)
C4—C5—C6—C7	−172.4 (4)	N1—C8—C9—O5	−1.1 (5)
C2—C1—C6—C5	−2.8 (6)	C8—N1—C11—C12	57.6 (5)
S1—C1—C6—C5	179.5 (3)	S1—N1—C11—C12	−85.1 (4)
C2—C1—C6—C7	172.8 (4)		

### Hydrogen-bond geometry (Å, °)

**Table:**

*D*—H···*A*	*D*—H	H···*A*	*D*···*A*	*D*—H···*A*
O3—H3···O4	0.82	1.92	2.628 (5)	144
C4—H4···O4^i^	0.93	2.55	3.395 (5)	152
C10—H10B···O2^ii^	0.96	2.52	3.320 (5)	141

Symmetry codes: (i) *x*−1/2, −*y*+1/2, *z*+1; (ii) −*x*+1, −*y*, *z*−1/2.
